# Bioinformatics analysis of neutrophil-associated hub genes and ceRNA network construction in septic cardiomyopathy

**DOI:** 10.18632/aging.206092

**Published:** 2024-08-30

**Authors:** Qingfei Cao, Jing Li, Meixue Chen

**Affiliations:** 1 Department of Urology, The First Affiliated Hospital of Jinzhou Medical University, Jinzhou, China; 2 Department of Pediatric, The First Affiliated Hospital of Jinzhou Medical University, Jinzhou, China

**Keywords:** septic cardiomyopathy, MRC1, neutrophils-related gene, biomarker, ceRNA network

## Abstract

Septic cardiomyopathy (SCM) is a critical sepsis complication characterized by reversible cardiac depression during early septic shock. Neutrophils, integral to innate immunity, can mediate organ damage when abnormal, but their specific role in sepsis-induced myocardial damage remains elusive. Our study focuses on elucidating the role of Neutrophil-Related Genes (NRGs) in SCM, finding early diagnosis and treatment biomarkers. We identified shared differentially expressed genes (DEGs) from datasets GSE79962 and GSE44363 and pinpointed hub DEGs using the cytoHubba plugin in Cytoscape software. The Neutrophil-Related Hub Gene (NRHG) MRC1 was identified via intersecting hub DEGs with NRGs from WGCNA. We validated MRC1's abnormal expression in SCM using our data and external datasets. Furthermore, a neutrophil-related ceRNA network (AC145207.5/ miR-23a-3p/MRC1) was constructed and validated. Our findings reveal MRC1 as a potential NRHG in SCM pathogenesis, offering insights into neutrophil-mediated mechanisms in SCM and providing a novel molecular target for early diagnosis and intervention in SCM.

## INTRODUCTION

Sepsis, a critical immune response dysregulation leading to organ dysfunction [1], has an incidence of 45 cases per 10,000 individuals and a mortality rate of about 20% [2, 3]. Septic cardiomyopathy (SCM), characterized by reversible cardiac depression in early severe sepsis and septic shock [4], significantly impacts patient outcomes [5]. The primary pathologic mechanisms in SCM involve cardiomyocyte damage and myocardial dysfunction. Currently, SCM diagnosis relies on myocardial damage markers and echocardiography [6, 7], limiting early patient intervention. Identifying novel biomarkers for SCM could be crucial in reducing mortality.

Non-coding RNAs (ncRNAs), unable to encode proteins, encompass both small non-coding RNAs (sncRNAs) and long non-coding RNAs (lncRNAs) [8]. MicroRNAs (miRNAs), a sncRNA type, along with lncRNAs, play pivotal roles in various physiological processes [9–11]. The competitive endogenous RNA (ceRNA) networks, comprising lncRNAs sharing miRNA response elements with mRNAs, regulate disease progression, including in sepsis and SCM [12, 13]. For instance, the lncRNA TTN-AS1/miR-29a/E2F2 ceRNA network has been implicated in SCM-related myocardial damage reduction [13].

In SCM, abnormal immune responses are a key factor in cardiomyocyte dysfunction. The innate immune system, including crucial neutrophil components, plays a vital role in sepsis progression [14–16]. Dysfunctional neutrophils may directly cause organ damage in sepsis [17], implicating their potential role in sepsis-induced myocardial damage. However, the exact mechanism remains unexplored.

**Figure 1:**
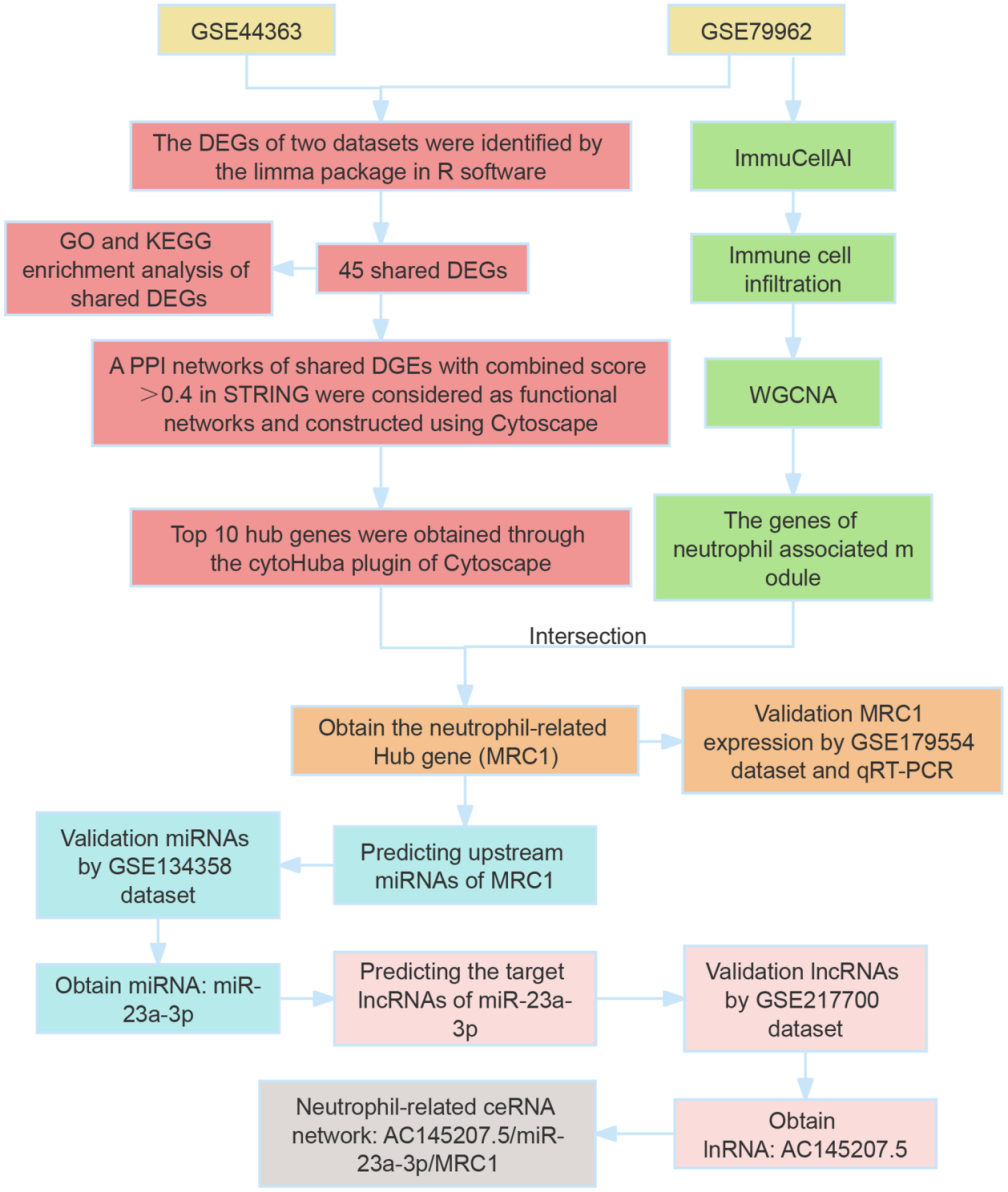
**Overview of study workflow.** This figure illustrates the sequential steps followed in the study, encompassing the identification of differentially expressed genes (DEGs), Gene Ontology (GO) and Kyoto Encyclopedia of Genes and Genomes (KEGG) analyses, and the construction of a protein–protein interaction (PPI) network.

Our study utilized bioinformatics analysis to identify differentially expressed genes (DEGs) in SCM, using datasets GSE79962 and GSE44363. We performed GO/KEGG enrichment analysis on the shared DEGs, followed by constructing a protein-protein interaction (PPI) network using the STRING database. The top 10 hub genes were identified using CytoHubba plugin, and variations in immune cell infiltration in SCM were analyzed with the ImmucellAI tool. We also employed weighted gene correlation network analysis (WGCNA) to identify neutrophil-related hub genes (NRHGs), which were further validated by RT-PCR and additional datasets. Subsequently, we determined upstream miRNAs and lncRNAs of NRHGs, leading to the construction of significant neutrophil-related lncRNA-miRNA-NRHGs ceRNA networks. The study’s workflow is summarized in Figure 1.

## MATERIALS AND METHODS

### Data sources and characteristics

In our study, we sourced five datasets from the Gene Expression Omnibus (GEO) database (https://www.ncbi.nlm.nih.gov/geo/) to investigate septic cardiomyopathy (SCM) in both humans and mice, specifically focusing on datasets GSE79962, GSE44363, GSE179554, GSE134358, and GSE217700. Table 1 in our manuscript provides a detailed overview of these datasets. All datasets can be searched and downloaded from the GEO website (https://www.ncbi.nlm.nih.gov/geo/). We conducted analysis on GSE79962 and GSE44363 to identify NRHGs and establish a corresponding ceRNA network. Subsequently, we utilized additional datasets (GSE179554, GSE134358, and GSE217700) for validation purposes, verifying the screened mRNAs, miRNAs, and lncRNAs in the context of SCM.

**Table 1:** Information of the GEO datasets.

**GEO accession**	**Experiment type**	**Species**	**Source tissue**	**Sample**	**Data**	**Attribute**	**Control**	**SCM**
GSE79962	Array	Human	Myocardial tissue	11	20	mRNA	Test set
GSE44363	Array	Mice	Myocardial tissue	4	4	mRNA	Test set
GSE179554	High-throughput sequencing	Mice	Myocardial tissue	4	4	mRNA	Validation set
GSE134358	Array	Human	Blood	82	158	miRNA	Validation set
GSE217700	High-throughput sequencing	Human	Blood	4	4	lncRNA	Validation set

### Screening DEGs

To identify DEGs in SCM and control groups, our analysis utilized the limma package in R software (version 4.1.3). We employed the Benjamini and Hochberg method to control the false discovery rate (FDR). DEGs were selected based on the criteria of an adjusted *p*-value of less than 0.05 and an absolute log2 fold change (|log2FC|) of 1.0 or more. The datasets GSE79962, GSE44363, and GSE217700 were processed to generate heatmaps and volcano plots using the pheatmap and ggplot2 packages in R, respectively.

### Enrichment analyses

To explore pathways associated with shared DEGs in both humans and mice, we analyzed shared DEGs from the GSE79962 and GSE44363 datasets using the Gene Ontology (GO) and Kyoto Encyclopedia of Genes and Genomes (KEGG) databases. This analysis was performed with the clusterProfiler package in R software. For visual representation of the results, the ggplot2 package was utilized. The threshold for statistical significance in these analyses was set at a *p*-value of less than 0.05.

### PPI analysis

To construct protein-protein interaction (PPI) networks [18], we employed the STRING database (https://cn.string-db.org/), setting a medium confidence threshold of 0.4. Subsequently, the PPI assessment results were imported into Cytoscape software [19] (version 3.8.2) for visualization and further analysis. Within Cytoscape, the cytoHubba plugin [20] was utilized, leveraging Maximal Clique Centrality (MCC) calculations, to identify and highlight hub genes within the biological network, thus facilitating a deeper understanding of the interplay among shared DEGs.

### Immune cell infiltrate analyses

To assess immune cell infiltration in the GSE79962 dataset, we utilized ssGSEA enrichment analysis through the ImmuCellAI online platform (https://guolab.wchscu.cn/ImmuCellAI/#!/) [21]. This analysis, based on the expression of immune cell-specific marker genes, allowed us to evaluate the presence of 24 different immune cell types in the GSE79962 dataset. We compared this to a pre-defined gene matrix of immune cells. To illustrate our findings, we employed the corrplot and vioplot packages in R software to create a correlation heatmap and violin plot, respectively. These visualizations effectively demonstrate variations in immune cell infiltration and the correlations across the 24 immune cell types.

### Identification of neutrophil-related modules by WGCNA

In our research, we employed Weighted Gene Co-expression Network Analysis (WGCNA) to categorize genes into modules correlating with specific phenotypes. Utilizing the WGCNA package in R, we constructed a weighted co-expression network from the GSE79962 dataset, ensuring data quality through the goodSamples Genes function. The selection of a minimal power for the soft threshold ensured a scale-free topological fit index of 0.9, enhancing the identification of strongly correlated genes. Genes were clustered into modules based on correlation, with subsequent grouping and merging using average linkage hierarchical clustering. Modules with over 120 genes were specifically noted. We then calculated correlation coefficients and *P*-values to discern modules significantly linked with neutrophil infiltration. A heatmap created using the pheatmap package in R depicted the relationship between various immune cells and gene modules.

### Cell culture and treatment

H9c2 rat cardiomyocyte cells, provided by Shanghai Zhong Qiao Xin Zhou Biotechnology Co., Ltd, were cultured in Dulbecco’s Modified Eagle’s Medium (DMEM) enriched with 10% fetal bovine serum (FBS). To simulate Septic Cardiomyopathy (SCM) *in vitro*, these cells were exposed to 10 g/mL Lipopolysaccharide (LPS) for 12 hours. A control group was treated with normal saline over the same duration. This approach facilitates the investigation of SCM mechanisms at the cellular level.

### Real-time PCR

Total RNA was isolated using the Bioteke Total RNA Rapid Extraction Kit. Reverse transcription was performed with Beyotime Biotechnology’s BeyoRT II M-MLV transcriptase. Real-time PCR employed SYBR Green (BioTeke, China) and 2Taq PCR MasterMix (Solarbio, China), conducted on an Exicycler 96 thermocycler (Bioneer, USA). Data analysis was executed using the 2^−ΔΔCt^ method, with glyceraldehyde-3-phosphate dehydrogenase (GAPDH) as the internal control for the target gene. Primer sequences used are detailed in Table 2 of our manuscript.

**Table 2:** Primer sequences.

Gene	Direction	Sequences
MRC1	Forward	5′-ATGGGCAACATCGAGCAGAA-3′
	Reverse	5′-AAACCAATGCAACCCAGTGC-3′
GAPDH	Forward	5′-GTTCCTACCCCCAATGTGTCC-3′
	Reverse	5′-TAGCCCAAGATGCCCTTCAGT-3′

### GSEA

To elucidate the biological functional implications of MRC1, we conducted Spearman correlation analysis between MRC1 and all genes within the GSE79962 dataset. The resulting correlation coefficients were then ranked and utilized for Gene Set Enrichment Analysis (GSEA), employing Gene Ontology (GO) gene sets sourced from the Molecular Signatures Database (MSigDB, https://www.gsea-msigdb.org/gsea/msigdb/index.jsp). The top 5 biological process (BP) enrichment results were subsequently visualized to highlight the most significant biological functional associations of MRC1.

### Prediction of target miRNAs

To identify the target miRNAs of our gene of interest, we utilized three prominent online databases: StarBase v2.0 (https://starbase.sysu.edu.cn/) [22], miRDB (http://mirdb.org) [23] and TargetScan (https://www.targetscan.org/vert_80/) [24]. The overlapping miRNAs predicted by these databases were identified through intersection analysis. We visualized this intersection using a Venn diagram to present the prediction results clearly. Further validation of these intersected miRNAs was carried out using data from the GSE134358 dataset, enabling us to pinpoint the target miRNAs with higher precision.

### Construction of ceRNA networks

To discover lncRNAs interacting with our target miRNA, we utilized StarBase v2.0 (https://starbase.sysu.edu.cn/) [22] and LncBase v3.0 (DIANA Tools - lncBase v.3 (uth.gr)) [25] databases. The intersection of predicted lncRNAs in Homo sapiens from these databases with the differentially expressed lncRNAs from the GSE217700 dataset led to the identification of target lncRNAs. These targets were further validated using the GSE217700 dataset. Subsequently, we constructed a competitive endogenous RNA (ceRNA) network using Cytoscape, illustrating the interactions among mRNA, miRNA, and lncRNA.

### Statistical analysis

For bioinformatics analyses in our study, R software (version 4.1.3) was employed. Statistical analyses comparing two groups were performed using GraphPad Prism 10.1, applying either the *t*-test or signed-rank test as appropriate. The results were presented as mean ± SD, with a *p*-value threshold of less than 0.05 considered indicative of statistical significance.

### Data availability statement

The datasets utilized for the analyses conducted in this study were sourced from publicly accessible databases. These datasets are available for retrieval from the following website: GEO Accession viewer (https://nih.gov).

## RESULTS

### Screening shared DEGs in SCM

**Figure 2:**
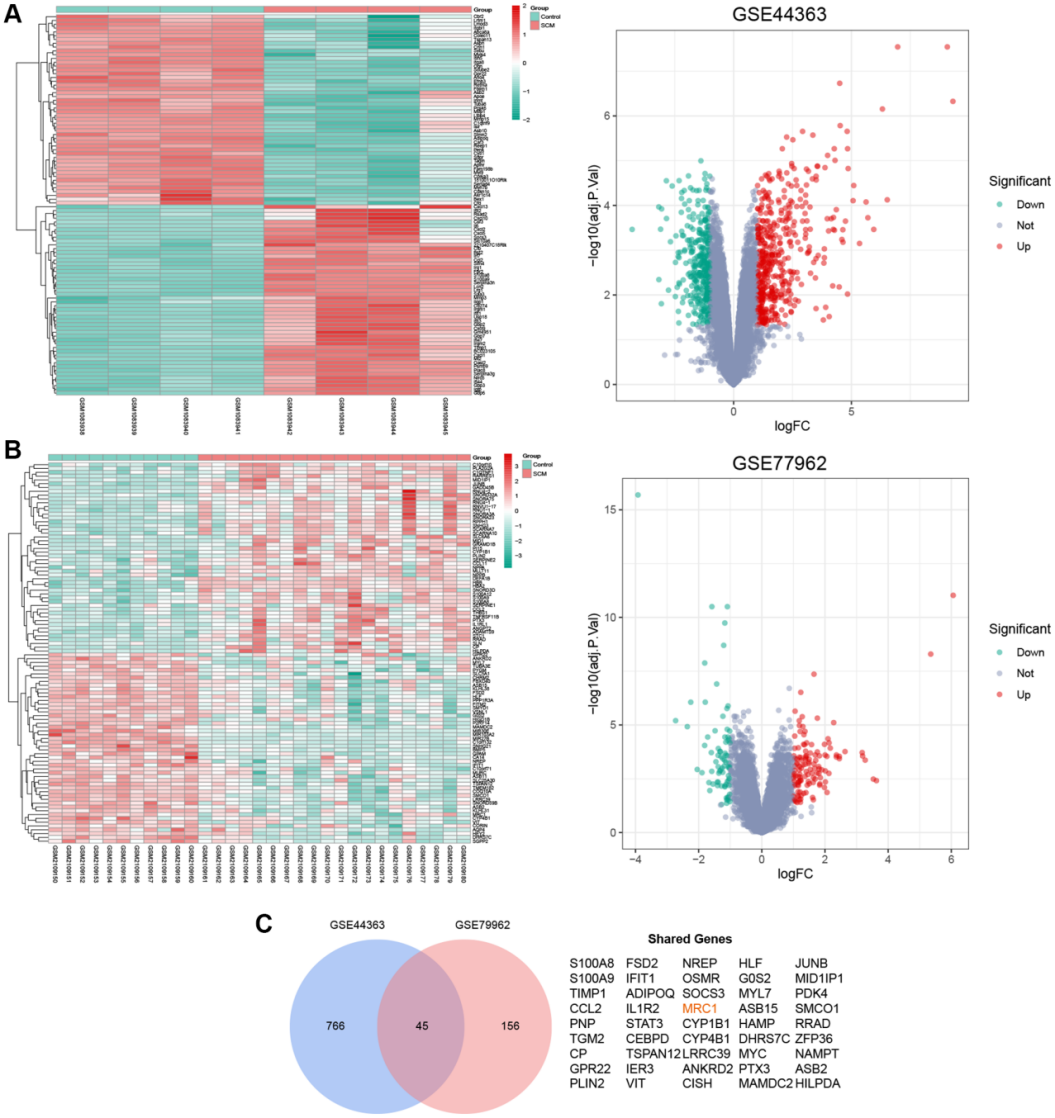
**Identification of shared DEGs in GSE44363 and GSE79962 datasets.** (**A**) Heatmap and volcano plot depicting the mRNA expression profile in the GSE44363 dataset. (**B**) Corresponding heatmap and volcano plot for the GSE79962 dataset. (**C**) Venn diagram highlights the 45 DEGs common to both datasets.

In our study, we analyzed two SCM gene expression profiles from the GEO database: GSE44363 (mouse-derived) and GSE79962 (human-derived). GSE44363 included data from 4 normal and 4 SCM mouse heart tissues, revealing 811 DEGs which 463 upregulated and 348 downregulated. GSE79962, comprising 11 normal and 20 SCM human heart tissues, identified 201 DEGs, with 125 upregulated and 76 downregulated. We created Supplementary Table 1 to detail these DEGs. Cluster heatmaps and volcano plots (Figure 2A, 2B) visually represent the data distribution for both datasets. A cross-analysis of these DEGs resulted in 45 shared DEGs (Figure 2C), which were selected for further investigation.

### Enrichment analysis of shared DEGs

**Figure 3:**
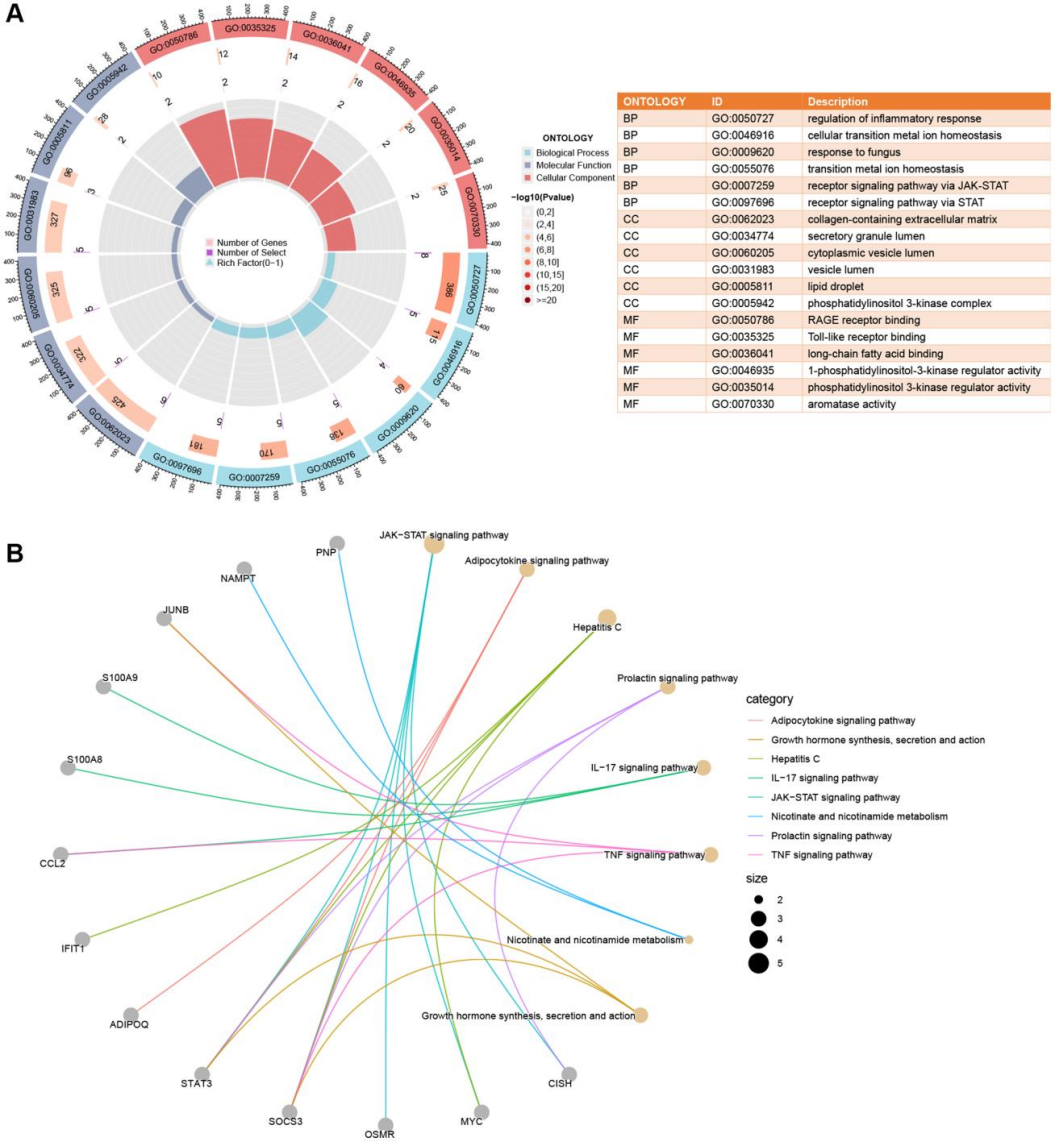
**Enrichment analysis of shared DEGs.** (**A**) A circle plot displays the top 6 enriched GO terms across Biological Process (BP), Cellular Components (CC), and Molecular Function (MF) categories. (**B**) Crosstalk analysis linking shared DEGs with KEGG pathways. Significance was determined using a Q-value threshold of < 0.05.

The shared DEGs in our study displayed significant enrichment across various biological processes, cellular components, and molecular functions. Notably, the top 3 biological processes included regulation of inflammatory response, cellular transition metal ion homeostasis, and response to fungus. For cellular components, the top 3 key areas were collagen-containing extracellular matrix, secretory granule lumen, and cytoplasmic vesicle lumen. In molecular functions, the top 3 prominent roles were RAGE receptor binding, Toll-like receptor binding, and long-chain fatty acid binding, as illustrated in Figure 3A. Additionally, KEGG enrichment analysis highlighted the JAK-STAT signaling pathway, Adipocytokine signaling pathway, and Hepatitis C signaling pathway as key pathways involved, detailed in Figure 3B.

### PPI network analysis and hub genes identification

**Figure 4:**
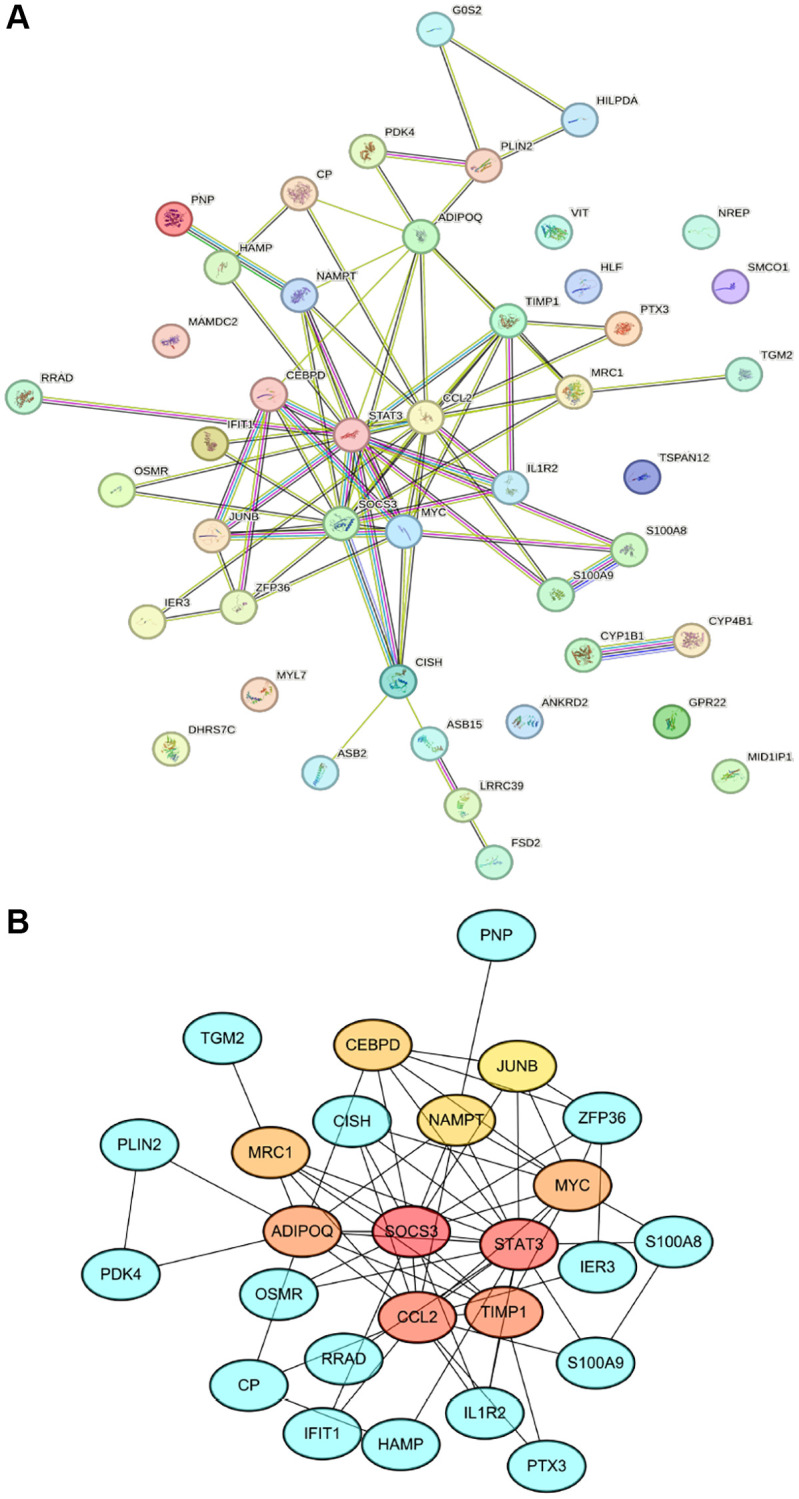
**PPI network construction and hub gene identification.** (**A**) Network diagram illustrating interactions between proteins encoded by shared DEGs, comprising 34 nodes and 75 edges. (**B**) Cluster plot highlighting the top 10 hub genes; the intensity of the node color correlates with the MCC score.

The interaction network, which maps the relationships between proteins encoded by shared differentially expressed genes (DEGs), was methodically constructed and visualized using the STRING online tool. This network is characterized by 34 nodes and 75 edges, as depicted in Figure 4A. Subsequently, we employed the Maximum Clique Centrality (MCC) algorithm of the cytoHubba plugin to identify the top 10 hub genes, illustrated in Figure 4B. These hub genes are hypothesized to have a significant impact on the pathogenesis of SCM, suggesting their critical role in the disease’s progression.

### The immune cell infiltration in SCM

**Figure 5:**
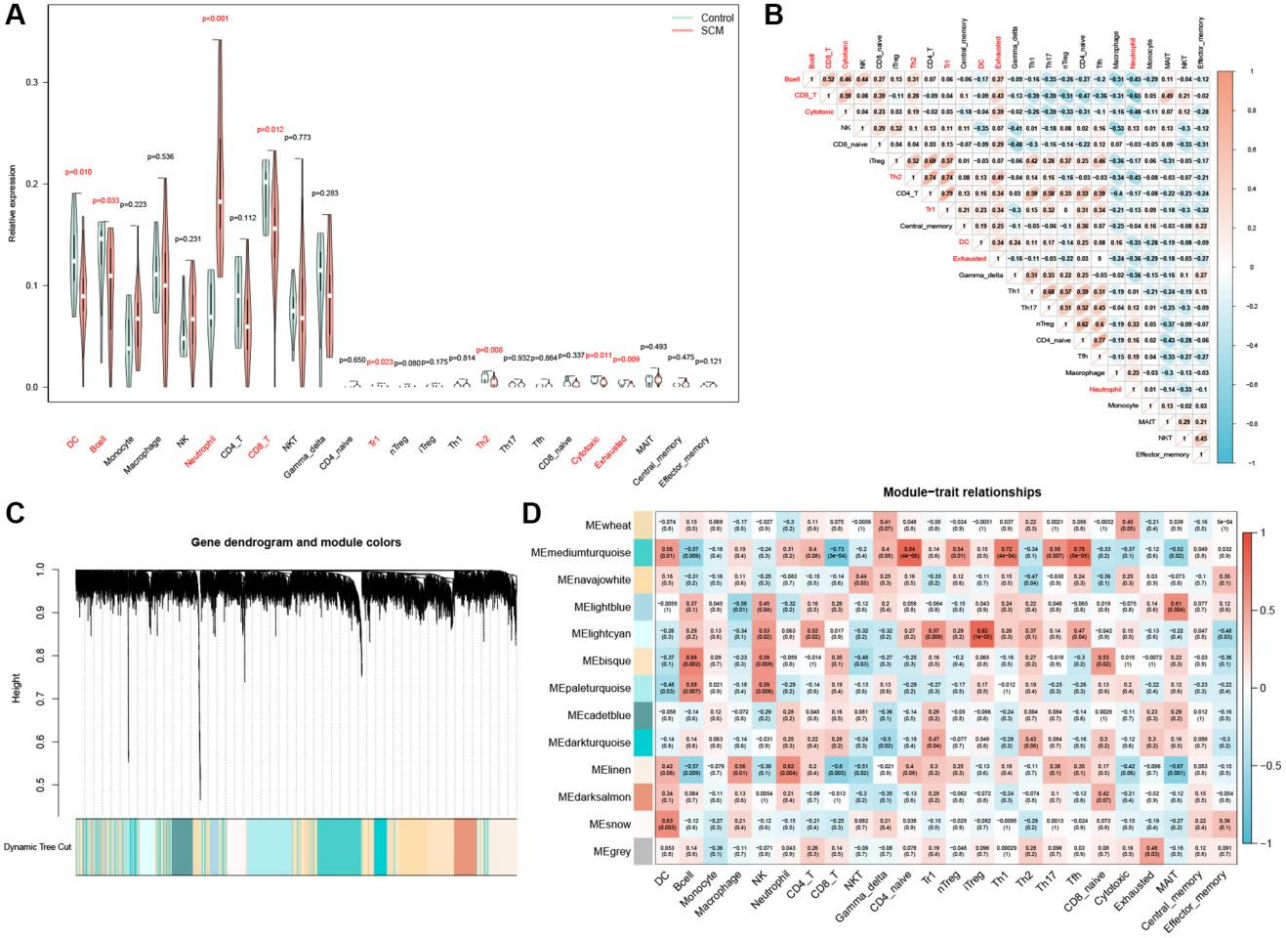
**Analysis of immune cell infiltration and module gene identification.** (**A**) Violin plot depicting the distribution of 24 immune cell types; significant differences between control and SCM groups are noted in red. (**B**) Heatmap of correlations between the 24 immune cells, with color coding indicating the nature of the correlation. (**C**) WGCNA-derived co-expression modules, displayed in a color-coded gene dendrogram. (**D**) Module-trait relationship grid, where each cell shows the correlation coefficient and *P*-value, with color indicating the direction of the correlation.

Initially, we imported the expression matrix of the GSE79962 dataset into the ImmuCellAI online tool. This process was aimed at determining the proportions of 24 types of immune cells, as detailed in Supplementary Table 2. Analysis via violin plots revealed a notable disparity in immune cell infiltration between the SCM and control groups. Specifically, neutrophil infiltration in the SCM group was significantly higher compared to the control group, as depicted in Figure 5A. Conversely, the presence of dendritic cells (DC), B cells, CD8+ T cells, Tr cells, Th2 cells, cytotoxic cells, and exhausted cells was markedly lower in the SCM group than in the controls (Figure 5A). Among these, neutrophils exhibited the most pronounced difference when compared to other differential immune cells, prompting us to select them for further analysis. Correlation heatmap further underscored this selection, demonstrating a significant negative correlation between neutrophils and various immune cells, including DC, B cells, CD8+ T cells, Th2 cells, cytotoxic cells, and exhausted cells (Figure 5B).

### Identification of NRGs by WGCNA

Utilizing WGCNA, we developed weighted gene co-expression networks based on the GSE79962 dataset. This process involved categorizing genes into distinct modules based on their correlation with 24 types of immune cells. Subsequently, we calculated and presented the correlation coefficients and *P*-values between each module and the infiltration of the 24 immune cell types in a heatmap format. Employing average linkage hierarchical clustering, 13 modules containing over 120 genes each were identified (as illustrated in Figure 5C). Notably, as depicted in Figure 5D, the linen module exhibited the highest correlation with neutrophil infiltration (r = 0.62, *p* = 0.004). Consequently, 804 genes within the linen module, detailed in Supplementary Table 3, were determined to have a strong association with neutrophil infiltration.

### Identification of NRHGs in SCM

**Figure 6:**
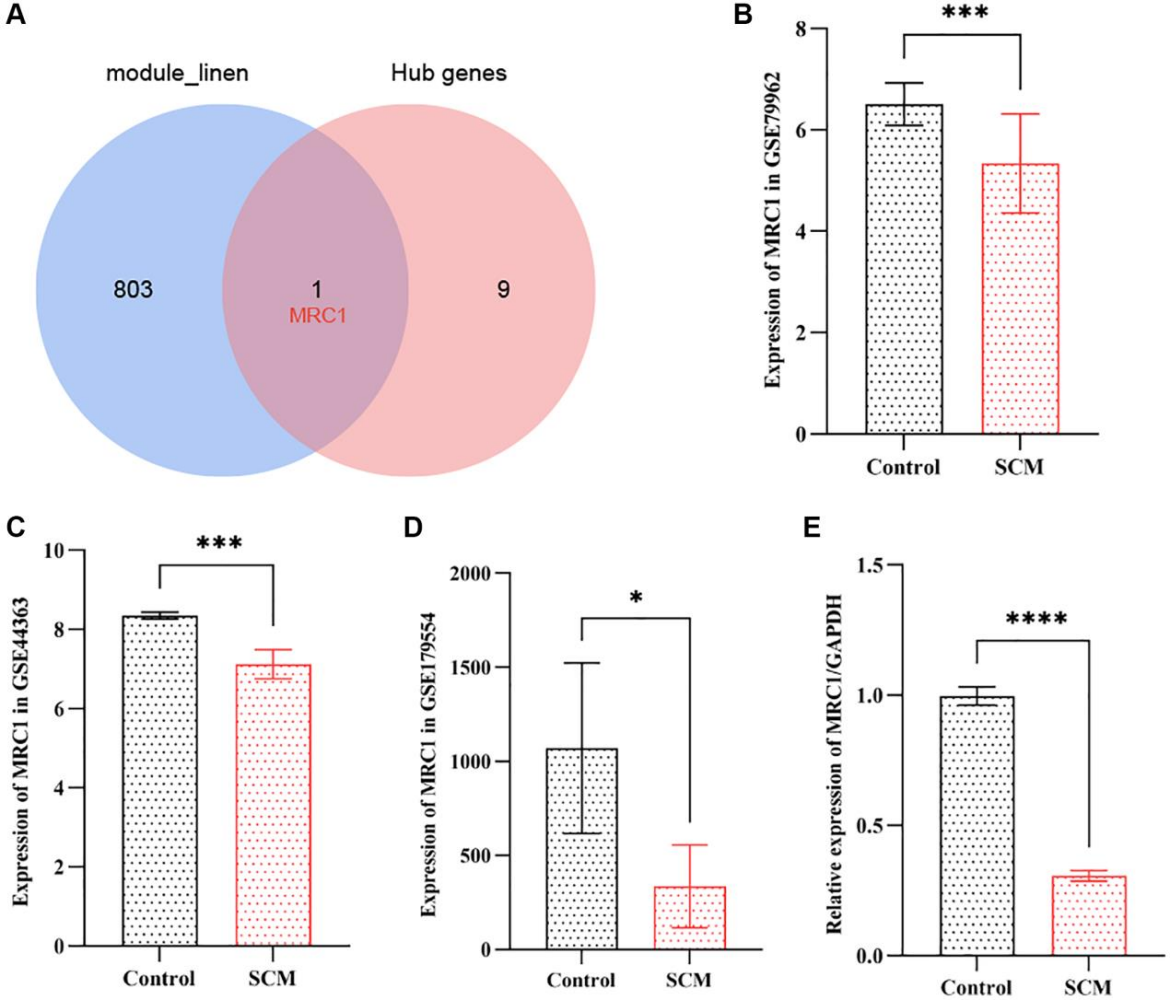
**Validation of neutrophil-related hub gene MRC1 in SCM.** (**A**) Venn diagram indicates intersection between top 10 hub genes and 804 neutrophil-related module genes. (**B**–**D**) Bar plots showing MRC1 expression levels in GSE79962, GSE44363, and GSE178554 datasets, respectively. (**E**) RT-PCR results illustrating significant downregulation of MRC1 expression. Significance levels are indicated (^*^p < 0.05; ^**^p < 0.01; ^***^p < 0.001; ^****^p < 0.0001).

In our study, we overlapped the top 10 hub genes with the 804 genes from the neutrophil-related module, leading to the identification of MRC1 as a key NRHG, as illustrated in Figure 6A. Subsequently, we extracted the expression levels of MRC1 from the GSE79962 and GSE44363 datasets and compared the differences between the SCM group and the control group using the *t*-test or signed-rank test, as appropriate. The results indicated that the expression of MRC1 was significantly lower in the SCM group compared to the control group in both datasets. Specifically, the GSE79962 dataset showed a *p*-value of less than 0.001, and the GSE44363 dataset showed a *p*-value of less than 0.01. These findings are illustrated in Figure 6B and 6C, respectively. To corroborate these findings, we examined the expression of MRC1 in the GSE179554 dataset using the same methodology. We observed a similarly significant reduction in MRC1 expression in the SCM group compared to the control group (*p* < 0.05), as depicted in Figure 6D. This trend was consistent with the findings from the GSE79962 and GSE44363 datasets. Further validation was conducted using RT-PCR on three samples of LPS-treated H9c2 cells and three samples of normal H9c2 cells. The results indicated that MRC1 expression in the LPS-treated group was significantly lower than in the control group (*p* < 0.0001), aligning with the trends observed in the public datasets, as shown in Figure 6E.

### Function of MRC1 in SCM

To further investigate the role of MRC1 in septic cardiomyopathy, we conducted a Spearman correlation analysis on the GSE79962 dataset, examining the relationship between the neutrophil-associated gene MRC1 and various immune cells. The results indicate that MRC1 is positively correlated with macrophages and negatively correlated with B cells and NK cells (Supplementary Figure 1A–1C). Additionally, GSEA analysis reveals that MRC1 is primarily enriched in the biological processes of complement activation, granulocyte activation, myeloid leukocyte activation, regulation of cell shape, and regulation of neutrophil activation (Supplementary Figure 1D).

### Prediction and validation of target miRNAs

**Figure 7:**
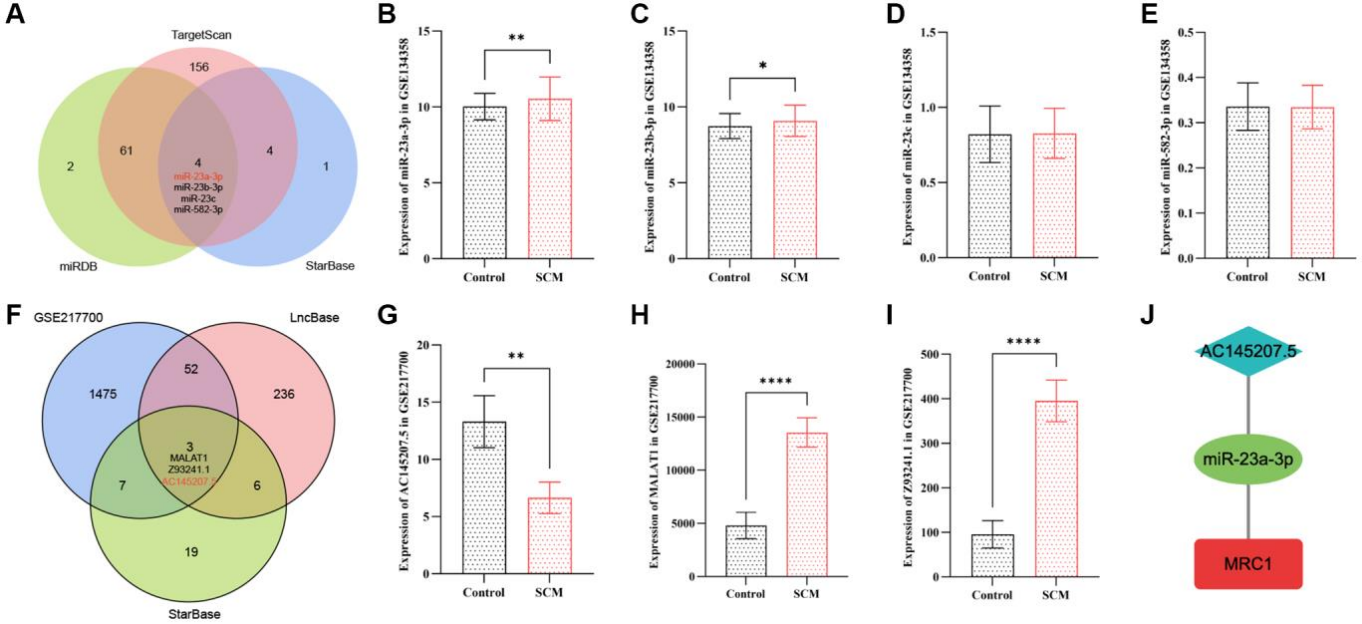
**Construction of the neutrophil-related ceRNA network AC145207.5/miR-23a-3p/MRC1.** (**A**) Venn diagram displaying intersecting miRNAs targeting MRC1, as predicted by three online databases. (**B**–**E**) Bar plots depict the expression of shared target miRNAs of MRC1 in the GSE134358 dataset. (**F**) Venn diagram shows intersecting lncRNAs targeting miR-23a-3p, as predicted by two online databases, with differential lncRNAs in GSE217700. (**G**–**I**) Bar plots for the expression of shared target lncRNAs of miR-23a-3p in GSE217700. (**J**) The constructed AC145207.5/miR-23a-3p/MRC1 ceRNA network. Significance levels are indicated (^*^p < 0.05; ^**^p < 0.01; ^***^p < 0.001; ^****^p < 0.0001).

In this study, we utilized three online databases: starBase v2.0, miRDB, and TargetScan, to predict microRNAs (miRNAs) targeting the MRC1 gene. The miRNA prediction results for MRC1 are delineated in Supplementary Table 4. By intersecting the prediction outcomes from starBase v2.0, miRDB, and TargetScan, we identified four common miRNAs: miR-23a-3p, miR-23b-3p, miR-23c, and miR-582-3p, as shown in Figure 7A. To further refine our search for the target miRNAs among these four candidates, we utilized the GSE134358 dataset to examine the expression differences of these miRNAs between the SCM group and the control group. The analysis revealed that only the expression levels of miR-23a-3p and miR-23b-3p differed significantly between the two groups. Notably, miR-23a-3p demonstrated the most pronounced difference, with an expression trend inversely correlated to that of MRC1. This observation aligns with the miRNA sponge effect, as depicted in Figure 7B–7E.

### Construction of neutrophil-related ceRNA regulatory network

Firstly, we employed two online databases, starBase v2.0 and lncBase v3.0, to predict lncRNAs interacting with miR-23a-3p. The results of these lncRNA predictions are comprehensively presented in Supplementary Table 5. Following this, we downloaded a dataset of sepsis lncRNA expression profiles, GSE217700, from the GEO database. This dataset, sourced from human peripheral blood, comprises 4 normal samples and 4 sepsis samples. From GSE217700, we identified 1537 differentially expressed lncRNAs (Supplementary Table 6), adhering to our criteria of an adjusted *p*-value < 0.05 and an absolute log2 fold change (|log2FC|) of at least 1.0. To further refine our analysis for lncRNAs interacting with miR-23a-3p, we intersected the predictions from the two databases with the differentially expressed lncRNAs in GSE217700, leading us to identify three target lncRNAs: MALAT1, Z93241.1, and AC145207.5, as shown in Figure 7F. Notably, among these lncRNAs, only the expression trend of AC145207.5 was in concordance with that of MRC1, as depicted in Figure 7G–7I. Finally, we constructed a neutrophil-related ceRNA network (AC145207.5/miR-23a-3p/MRC1) to elucidate the pathogenesis of SCM, using Cytoscape software, showcased in Figure 7J.

## DISCUSSION

SCM is a reversible form of cardiac depression occurring in the early stages of sepsis, predominantly characterized by left heart dysfunction [4, 26]. SCM is regarded as one of the most severe complications of sepsis, contributing to 18–65% of all sepsis-related complications. The global mortality rate associated with SCM ranges between 36–55%, approximately 2–3 times higher than that of sepsis alone [2, 5]. The immune system, encompassing both innate and adaptive immunity, plays a pivotal role in the progression of sepsis [14, 15]. Previous research has established the critical involvement of immune responses in the pathophysiology of SCM [27]. Neutrophils, as integral components of innate immunity, are key players in inflammatory-mediated organ dysfunction, often leading to direct organ damage [16, 17]. Previous studies have demonstrated that neutrophils play distinct roles at various stages of sepsis. During the early phase of sepsis, neutrophils play a critical role in combating bacteremia and maintaining immune homeostasis. However, in the later stages, the excessive infiltration of neutrophils often results in tissue damage and organ dysfunction [28]. Thus, understanding the function and status of neutrophils is vital for the effective treatment of sepsis and the prevention of organ dysfunction, including myocardial injury. In our study, utilizing the ImmuCellAI online tool, we analyzed immune cell infiltration in myocardial tissues from patients with septic cardiomyopathy within the GSE79962 dataset. Our findings indicated a significantly higher concentration of neutrophils in SCM tissue compared to the control group. Moreover, correlation analyses revealed that neutrophil levels were inversely associated with other immune cells, including dendritic cells (DC), B cells, CD8+ T cells, and Th2 cells. These observations align with previous studies suggesting that excessive neutrophil infiltration or dysfunction may contribute to myocardial damage in sepsis. However, the specific underlying biological mechanisms remain to be elucidated.

To identify NRGs intimately linked with SCM, our approach involved the use of Weighted Gene Co-expression Network Analysis (WGCNA) to isolate neutrophil-related gene modules within the GSE79962 dataset. Subsequently, PPI network was constructed using the STRING online database for shared DEGs. Employing the cytoHubba plugin, we pinpointed the top 10 hub genes: SOCS3, MYC, MRC1, ADIPOQ, CEBPD, JUNB, CCL2, STAT3, TIMP1, and NAMPT. By intersecting these hub genes with neutrophil-associated module genes, we identified NRHG-MRC1. The MRC1 gene, encoding the Mannose Receptor C-Type 1 or CD206 receptor, is situated on human chromosome 10. Functionally, MRC1 is adept at recognizing and binding various glycosylation fractions, thereby playing a significant role in immune response modulation and pathogen elimination [29, 30]. Predominantly expressed in dendritic cells and macrophages, MRC1 is implicated in processes such as inflammation, wound healing, and tumor-associated macrophages [31]. These results also confirm our findings that MRC1 is involved in various immune response processes. Previous studies have shown that neutrophils are activated and exhibit increased cell adhesion when stimulated by the CXCL4 immune complex. During this activation process, MRC1 is overexpressed in neutrophils, influencing their activation status and function, and playing a crucial role in the immune response of neutrophils [32]. Based on our conclusions regarding the biological processes of MRC1 enrichment in neutrophil activation, we believe that MRC1 plays a significant role in the inflammatory process of SCM. MRC1 shows potential as a novel diagnostic marker and therapeutic target for NRG in SCM. Although the role of MRC1 in the pathogenesis of SCM is acknowledged, the factors influencing its expression within this context remain undefined. The concept of the ceRNA network presents a novel mechanism for regulating gene expression [33]. To date, no ceRNA network specifically associated with neutrophils has been established in the study of SCM. Consequently, we ventured to construct a ceRNA network targeting MRC1. This initiative aims to elucidate the regulatory mechanisms of MRC1 in modulating neutrophil infiltration in SCM, potentially unveiling new insights into the disease's molecular underpinnings.

### AC145207.5/miR-23a-3p/MRC1 axis

Upon identifying MRC1 as a NRHG and confirming its targeted miRNAs through online prediction tools, we validated miR-23a-3p using public databases. Given that miRNAs primarily function to inhibit mRNA expression and promote mRNA degradation [34], their expression trends typically oppose those of mRNAs. In our study, the downregulation of MRC1 in SCM suggests an upregulation of upstream miRNA expression, aligning with the observed expression patterns of miR-23a-3p in the GSE134358 dataset. Notably, research by Toktam Moghiman et al. demonstrated that miR-23a-3p secreted by exosomes can mitigate myocardial ischemia [35]. Furthermore, several studies, including one that revealed PVT1 exacerbates cardiomyocyte death via the miR-23a-3p/CASP10 axis [36], and another by Dishiwen Liu et al., showing miR-23-3p’s role in promoting ferroptosis in cardiomyocytes [37], underscore the significance of miR-23a-3p in cardiac pathology. Complementing these findings, Vasundhara Kain et al. conducted a transcriptome analysis of leukocytes (macrophages/neutrophils) from infarcted left ventricles in a 12/15LOX-/- mouse model. Their results highlighted the elevated expression of EP4 on MRC1-expressing repair macrophages, with the deletion of 12/15LOX downregulating miR-23a-3p expression and fostering macrophage polarization towards a repair phenotype [38]. This evidence collectively supports our findings, suggesting a critical interaction between miR-23a-3p and MRC1 in myocardial infarction.

Further leveraging the capabilities of StarBase v2.0 and LncBase v3.0 online databases, we screened for lncRNAs capable of specifically binding to miR-23a-3p. This led to the identification of three lncRNA (MALAT1, Z93241.1, and AC145207.5) through intersection with differentially expressed lncRNAs in the GSE217700 dataset. Subsequent validation using the GSE217700 dataset confirmed the expression trends of these lncRNAs, culminating in the selection of lncRNA AC145207.5, which demonstrated a similar expression pattern to MRC1. Previous studies have shown that AC145207.5 and miR-23a-3p can form a ceRNA network in brain tissue [39]. AC145207.5, also known as RP11-498C9.15, has been primarily investigated in the context of prognostic model establishment for tumor patients and the assessment of immunotherapy efficacy [40–42]. Recent research also highlights AC145207.5’s crucial role in the pathogenesis of rheumatoid arthritis through the modulation of microRNA and gene expression [43]. In line with this, Wang Wenwen et al. demonstrated that the AC145207.5/miR-101-3p axis can impede immune cell infiltration by upregulating CAMSAP1 expression, contributing to adverse outcomes in advanced hepatocellular carcinoma patients [44]. These findings resonate with our observations and underscore the significance of AC145207.5 in the processes of immune cell infiltration and immune regulation.

The AC145207.5/miR-23a-3p/MRC1 axis may play a pivotal role in SCM by regulating multiple key processes. Neutrophils are crucial in the immune response during sepsis, and the ceRNA network involving AC145207.5 and miR-23a-3p modulates MRC1 expression [38, 39]. This regulation influences neutrophil-mediated functions such as phagocytosis and pathogen clearance, thereby impacting the inflammatory response in SCM [45]. miR-23a-3p, a known regulator of cytokine expression, is modulated by AC145207.5 acting as a ceRNA [39, 46]. This interaction potentially affects cytokine and chemokine production, further influencing the inflammatory milieu during SCM [46]. Moreover, MRC1 is associated with alternatively activated (M2) macrophages, which contribute to tissue repair and anti-inflammatory responses [47]. The ceRNA network may alter macrophage polarization, potentially shifting the balance between pro-inflammatory (M1) and anti-inflammatory (M2) macrophages in SCM. Additionally, miR-23a-3p targets genes involved in apoptosis [37], suggesting that AC145207.5 regulation could impact these pathways, ultimately affecting cardiomyocyte viability during SCM.

## CONCLUSION

In this study, we utilized GEO datasets from human and mouse samples to identify excessive neutrophil infiltration in septic cardiomyopathy (SCM) and, for the first time, uncovered the critical role of NRHG-MRC1 in SCM. Through external dataset analysis and RT-PCR validation, we confirmed the expression level of MRC1. A significant milestone of our research is the construction of a novel neutrophil-associated ceRNA network centered around MRC1, specifically AC145207.5/miR-23a-3p/MRC1. In summary, this study provides insights into the gene interactions within the AC145207.5/miR-23a-3p/MRC1 ceRNA network, highlighting the potential application value of MRC1 and its related network in SCM, and lays a foundation for future research.

## Supplementary Materials

Supplementary Figure 1

Supplementary Table 1

Supplementary Table 2

Supplementary Table 3

Supplementary Table 4

Supplementary Table 5

Supplementary Table 6
