# Supplementary material for: Bioinformatics analysis of neutrophil-associated hub genes and ceRNA network construction in septic cardiomyopathy
Source: Aging (Albany NY). 2024 Aug 30;16(19):12833–49. doi: 10.18632/aging.206092 (PMC11501391; doi:10.18632/aging.206092)
Supplement: Supplementary Figure 1 [file aging-16-206092-s001.pdf]

SUPPLEMENTARY FIGURE

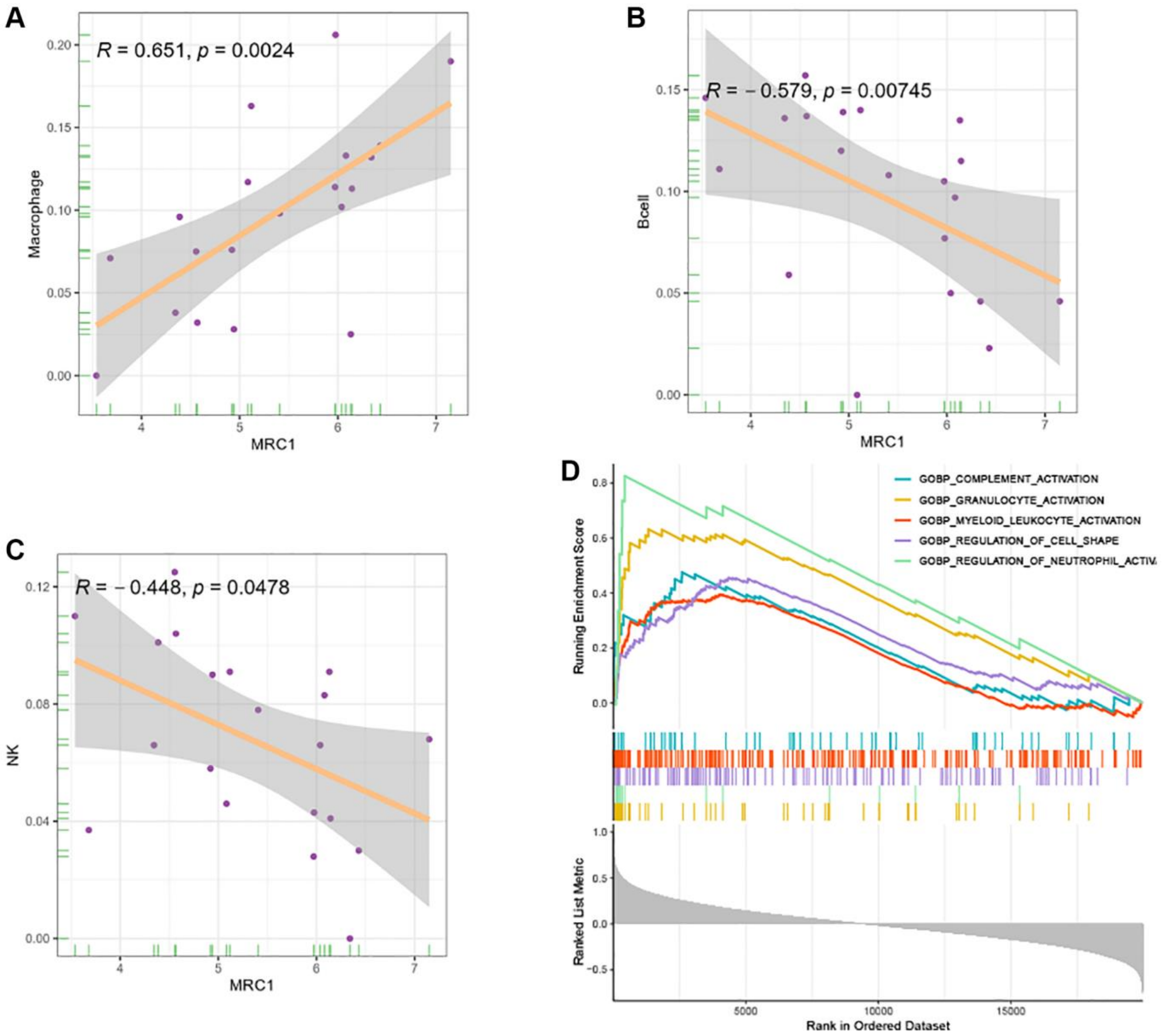

Supplementary Figure 1. Function of MRC1 in SCM. (**A**) Scatterplot of MRC1 correlation with macrophages. (**B**) Scatterplot of MRC1 correlation with B cells. (**C**) Scatterplot of MRC1 correlation with NK cells. (**D**) Top five GO BP associated with MRC1. Abbreviation: GO: Gene Ontology; BP: biological process; GSEA: Gene Set Enrichment Analysis.
